# Hematocrit alterations and its effects in naturally infected indigenous cattle breeds due to *Trypanosoma* spp. on the Adamawa Plateau - Cameroon

**DOI:** 10.14202/vetworld.2015.813-818

**Published:** 2015-06-30

**Authors:** A. Mamoudou, V. K. Payne, S. L. Sevidzem

**Affiliations:** 1 Department of Parasitology and Parasitological Diseases, School of Veterinary Medicine and Sciences, University of Ngaoundere, Cameroon; 2 Department of Animal Biology, Faculty of Science, University of Dschang BP 67 Dschang, Cameroon

**Keywords:** hematocrit, buffy coat method, body condition, anemia, trypanosome, breed, cattle, Adamaoua Plateau - Cameroon

## Abstract

**Aim:**

An experimental study was carried out on 148 naturally infected indigenous cattle breeds with either single or mix infections of various species of trypanosomes. The objectives of this study were to determine the species of trypanosomes, observe their hematopathological consequences on host-related risk factors and to determine the packed cell volume (PCV) of the infected group.

**Materials and Methods:**

The buffy coat method (BCM) which is a variant of the hematocrit centrifugation method was used for the parasitological and hematological analysis. The May Grünwald-Giemsa method was also used for the identification of different trypanosome species.

**Results:**

The infection rate in accordance with the various trypanosomes was as follows: *Trypanosoma congolense + Trypanosoma brucei* (1.35%), *Trypanosoma vivax* + *T. brucei* (1.35%), *T. congolense* + *T. vivax* (8.11%), *T. congolense* + *T. vivax* + *T. brucei* (8.78%), *T. brucei brucei* (11.48%), *T. vivax* (20.94%), *T. congolense* (47.97%). The infection rate with respect to breeds showed the following results - Brahman (1.0%), Red Fulani (5.2%), White Fulani (6.5%) and Gudali (16.7%), with no statistical significant difference (p>0.05). The combined mean PCV of single as well as mix infections was not statistically significant (p>0.05). The mean PCV of males (25.64±5.08 *standard deviation* [SD]) which was lower than that of females (30.82±4.94 SD) was statistically significant (p<0.05). The body condition of infected animals with sex showed that a greater proportion of males with “Poor” and “Medium” conditions showed high prevalence than females with the same conditions, with a significant difference (p<0.05). However, females showed a “Good” condition than males even though it was not statistically significant (p>0.05). The PCV profile of the infected group showed that the highest proportion of infected animals had PCV of ≤31% than PCV >31%. The mean weight of the animals was (265.41±95.36 SD). A scatter-linear plot of infected buffy coat against mean PCV showed a negative parametric correlation.

**Conclusion:**

Distinguished *Trypanosoma* spp. pathogenicity, emaciation and weight loss related anemia, poor body condition, sex and the response of different breeds to various trypanosomes were highly affected and are of vital importance in diagnosis and act as a contribution to future control and treatment plans in this area.

## Introduction

Cattle trypanosomiasis is an acute or chronic infectious disease caused by blood parasites belonging to the genus *Trypanosoma* [1], with size 10-40 µm and 1.5-3 µm in width. The most common trypanosome species and subspecies in this area include: *Trypanosoma congolense*, *Trypanosoma vivax* and *Trypanosoma brucei brucei*. These trypanosomes are transmitted mechanically and biologically by infective bites of tsetse fly (genus: Glossina) [2], and other hematophagous Diptera of genera Tabanus, Stomoxys, Hematopota and Lyperosia. The three northern regions: Adamawa, North and Far North, are considered to be the major cattle producing areas in Cameroon. The Adamaoua, in particular, is the highest cattle rearing region as it harbors 28% of the estimated 10 million Cattle in Cameroon and contributes to about 38% of beef production in the country [3].

This disease, colloquially known differently in this region as “Piale badawde” and “Piale boubi,” is a menace to livestock and agriculture in the area. The livestock/wildlife ecological zone of Alme in the Adamaoua Plateau serves as a touristic ground, but the disease is a threat to the lives of animals found in the natural game reserves in the area such as in the Faro Game Reserve, Alme Park and Gashaka Park, which border this area. The availability of abundant pasture lands is of nutritional importance to the sedentary herds in the area, which have increased from 5 [4] to more than 300 herds today, partly due to transhumance. Good nutritional status, coupled with the fact that these animals have been co-habiting for several millennia has rendered the disease less severe and animals are immuno-tolerant [5].

Anemia as indicated by the PCV of infected animals is very important in the symptomatology of blood scavenging parasites. Anemia-related trypanosomiasis is caused by extra-vascular hemolysis through erythrophagocytosis in the mononuclear phagocytic system of the spleen, liver and hemal nodes [6,7]. Decreased blood volumes caused by trypanosomes result in opportunistic helminthosis [8], which severely affects the overall livestock production in a purely livestock mix farming system typical of this area.

The pathological consequences become severe depending on the trypanosome species and sub-species, within a livestock, between livestock species and among breeds depending on the challenge and virulence of the strains [9,10]. Due to ignorance of the symptoms, signs and pathology of the disease, farmers tend to treat these animals with trypanocides without prior laboratory diagnosis and confirmation. Due to the financial constraint of farmers, they under dose the animals or sometimes administer wrong therapy with fake drugs, which have contributed to a widespread trypanocidal drug resistance [11].

While waiting for a reliable vaccine for bovine trypanosomiasis, several research projects should be focused on this disease to add to the few preliminary works already achieved. Our present study seeks to know the consequences of hematocrit alterations caused by bovine trypanosome species in naturally infected indigenous cattle breeds in the tsetse infested zone (Alme) of the Adamaoua Plateau of Cameroon, with the objectives of identifying the various trypanosome species, their effects on mean PCV, on different cattle breeds and on other host related risk factors, as an integrative contribution to diagnosis of the disease, control and treatment plans in this area.

## Materials and Methods

### Ethical approval

Necessary permissions from the Faculty of Science of the University of Dschang, Cameroon were taken to conduct the research.

A verbal consent was demanded from the herders and cattle owners, with care taken in blood collection in order not to harm the animals. Motivations were made, which also included treatment of sick animals after the study.

### Study area

**Figure-1:**
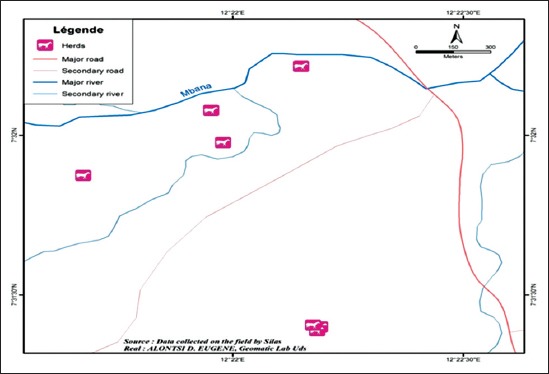
Map showing sampled herds of study animals.

Moving further North of Cameroon, the climate changes to the Guinean, the Sahelo-sudanian and to Sahelian in the Far north. There is only one rainy season from April to October in the Guinean zone and in the Sahelian zone it is rather from June to September. Annual rainfall ranges from 1400 to 1700 mm in the Guinean zone, 800-1400 mm in the Sahelo-sudanian zone and around 600 in the Sahelian zone [12]. The Faro and Deo Division is remarkable in the history of the tsetse eradication Campaign. This Division was stratified by the Special Mission for the Eradication of Glossines after the 1994 eradication campaign into: Plateau (non-infested zone), Buffer zone which acted as a barrier to tsetse re-invasion from the valley (infested zone) to the Adamaoua Plateau. Our study area (Alme) is found in the tsetse infested zone of the Faro and Deo Division of the Adamaoua Plateau. It is a livestock/wildlife ecological as well as an agricultural zone, located between Latitude 7° North and Longitude 12° East (Figure-1). This area is covered with savannah type vegetation, more than 90%, which consist of *Daniella olivert* and *Lophira lanceolata* [4]. This study area is characterized by abundant pastoral land for the available greater than 20 sedentary and semi-sedentary herds, available at the time of study. Hence good nutritional status of cattle in this area.

A one-off sampling was conducted on naturally infected adult cattle in Alme Village (n=148). Selection of these animals was based on logical reasoning, with only infected animals considered and other related factors such as sex and breed considered as criteria. The animals were allowed to graze freely in the area with communal herding in a traditional husbandry system, with no treatment effected in the course of the study. All the animals detected with trypanosomes were appropriately treated intra-muscularly (IM) with trypanocides such as Veriben B12^®^ (Ceva, France) at a curative dose of 7 mg/kg body weight. A pour-on product known as vectochlor^®^ and cypermethrin, chlorpyrifos, piperonyl butoxide and citronel, was also applied on these sick animals.

### Physical examination of the Animals

The body condition and weight of these animals were recorded. Body condition is an easy and more economical way to evaluate the body fat percentage of cattle. The commonly used scale of 1-9 with 1 representing an emaciated case and 9 an obese case [13]. It is a very cheap and easily manipulated picture-charted semi-quantitative method for the determination of health status of cattle. It considers important anatomical landmarks, such as bony prominence of the chest region, spinous processes and back bones (hip and pin), which enabled us to group the animals into “Good,” “Medium” and “Poor” conditions.

The weight of animals were measured in order to confirm the health status (body condition) of these animals, using a 20 m 50 zoo meter belt and values were keyed and computed using the Shaeffer’s formula for the prediction of body weight of cattle [14] as shown below:


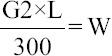


Where:

W=Body weight of the animal

G=Heart girth

L=Length from the point of shoulder to the point of hip bone.

### Hematocrit determination

Whole blood was collected by veino-puncture into ethylene diamine tetra-acetate anticoagulated tubes. Micro-capillary tubes were then charged up to ¾ of their height, then sealed with “cristaseal” (Hawksley, Lancing UK) and centrifuged at 12000 rpm for 5 min using the Hawksley hematocrit centrifuge. The PCV of the animals was then estimated using a Hematocrit reader. Packed Cell Value readings below 31% were considered as anemic.

### Trypanosome detection

Micro-capillary tubes were cut 1mm below the buffy coat zone. The buffy coat was then extruded onto a glass slide and covered with a 22 mm ×22 mm cover slide and examined using dark-ground microscopy [15]. Further confirmation of positive samples and detail morphological examination was done using the May-Grunwald Giemsa staining technique described by Benjamin [16]. Species and subspecies of trypanosomes namely: *T. congolense*, *T. vivax, T. brucei brucei* were identified as described by Soulsby [17].

### Statistical analysis

The statistical software used in data analysis was SPSS version 19.0. The Chi-square test was used to compare the various body conditions with sex and also the infection rate with the breed. One-way ANOVA was used to compare the mean PCV with trypanosome species and MS-Excel version of windows 2007 was used to bring out the parametric relation of infection rate and mean PCV of the experimental group.

## Results

### Relation between mean PCV and trypanosome species (single and mix)

Single as well as mix infections caused a reduction in PCV in the animals. It was interesting to know that the difference in the reduction of this PCV was trypanosome species dependent. *T. congolense* + *T. brucei* mix infection showed the least PCV reduction (23±7.07 SD) followed by *T. congolense* single infection (26.97±5.61 SD), but no statistically significant difference (p>0.05) (Table-1). In addition, prevalence and PCV trends relative to *Trypanosoma* spp. revealed that *T. congolense* (prevalence=47.94%; PCV=26.97%) was most prevalent and pathogenic among singly infected animals, while *T. congolence* + *T. brucei* (prevalence=1.35%; PCV=23%) was least prevalent and most pathogenic among mix infections in this area (Table-1).

**Table-1:** The mean PCV of infected animals with trypanosome species.

Species	I/E	Prevalence (%)	Mean PCV±SD	p value
Tc	71/148	47.94	26.94±5.61	
Tv	31/148	20.9	28.13±3.71	
Tb	17/148	11.48	27.76±5,32	
Tc+Tv	12/148	8.11	29.08±8.55	
Tc+Tb	2/148	1.35	23±7.07	
Tv+Tb	2/148	1.35	31±1.41	
Tc+Tv+Tb	13/148	8.78	28.77±6.85	0.598

Tc=*Trypanosoma congolense*, Tv=*Trypanosoma vivax*, Tb=*Trypanosoma brucei*, I=Number in infected, E=Number examined, SD=Standard deviation, PCV=Packed cell volume

### The PCV profile of infected animals

**Figure-2:**
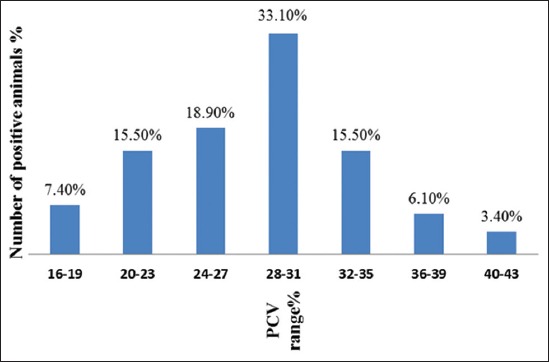
Packed cell volume profile of the animals.

The PCV profile of this group of animals revealed that a high proportion of infected animals 33.10% fell within the PCV interval of (28-31%) while the least fraction 3.40% fell within (40-43%) meaning that the mean PCV of less than 31% indicated anemia (Figure-2).

### Comparison of mean PCV with sex

The infection rate with sex showed that males (91/148, 61.49%) had a high infection rate than females (57/148, 38.51%) which also reflected in their difference in mean PCV values (25.64±5.08 SD and 30.82±4.94 SD respectively) was also statistically significant (p<0.05) (Table-2).

**Table-2:** Comparison of mean PCV with sex.

Sex	Mean PCV±SD	Number infected (%)	t-test	p value
Male	25.64±5.08	91 (61.49)		
Female	30.82±4.94	57 (38.51)		
Total	27.64±5.62	148 (100)	6.101	0.000

PCV=Packed cell volume, SD=Standard deviation

### Considered risk factors

**Figure-3:**
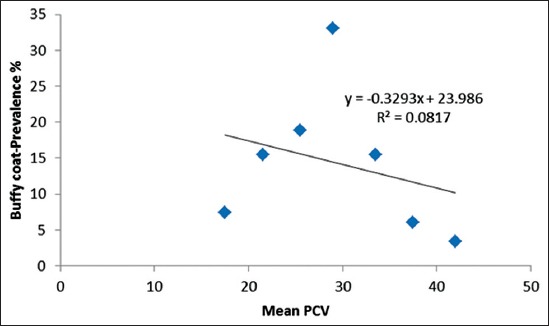
Scatter-linear plot of mean packed cell volume and buffy coat technique prevalence.

Body condition, breed susceptibility and body weight were considered as risk factors in the study. Concerning body condition, a greater fraction of “Poor” and “Medium” body condition male animals were infected than females with the same conditions with a statistical significant difference (p<0.05), but females were in a slightly “Good” condition than males with no statistical difference (Table-3). Meaning that female animals looked generally good than males. As concerns the infection rate with respect to various breeds- Brahman (3.38%), Gudali (56.76%), Red Fulani (17.57%) and White Fulani (22.30%). The Brahman race showed the least infection rate in the area while Gudali showed highest infection rate, but it was ironical that these differences in breed susceptibility did not show any statistical significant difference (p>0.05) (Table-4). The mean weight of the infected cattle breeds was 265.41±95.36 SD. The association of mean PCV with infection rate (Figure-3) showed that the mean PCV decreased with increase proportion of the positive buffy coat.

**Table-3:** Comparison of body condition score of infected cattle with sex.

Sex	Body condition score		

	Poor	Medium	Good
Male	49^a^	34^a^	8^a^
Female	28^b^	19^b^	10^a^

Each uniform subscript letter (a, b) denotes categories whose column proportions do not differ significantly from each other at the 0.05 level but those with non-uniform letters differ significantly at the 0.05 level.

**Table-4:** Susceptibility of cattle breeds to infection with trypanosomes.

Breed	Number positive	Prevalence	*χ*^2^	p value
Brahman	5	3.38		
Gudali	84	56.7		
Red Fulani	26	17.57		
White Fulani	33	23.3	2.33	0.506

*χ*^2^=Chi-square

## Discussion

**Figure-4:**
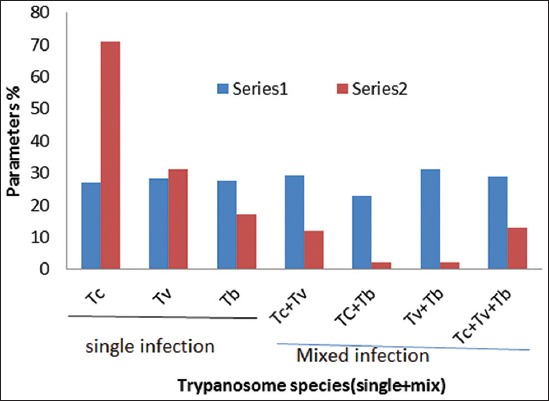
Packed cell volume and infection rate trends with parasitism (single + mix trypanosome infections). Tc=*Trypanosoma congolense*, Tv=*Trypanosoma vivax*, Tb=*Trypanosoma brucei*.

The trend of mono-specific, bi-specific, and tri-specific trypanosome parasitism with hematocrit and prevalence can be easily appreciated (Figure-4). This is important in the prediction of species-specific pathogenicity, vector types, and the epizootiology of disease in the area. Hemopathological consequences observed can be likened to the fact that, the mean PCV of the animals were generally reduced by *T. congolense* + *T. brucei* with high prevalence among mix infections, this finding corroborates with that of [18]. With respect to single infections, *T. congolense* infection showed a comparatively lower hematocrit value and highest prevalence, this finding corroborates with that reported by Tasew and Duguma [10] revealing that the bi-parasitic and mono-parasitic infections of these parasites were severe, as compared to the rest of the mix and single infections.

The highest prevalence and low hematocrit recorded with *T. congolense* (prevalence=47.94%; PCV=26.97%) than other single infections in the area, indirectly indicates the superiority of tsetse flies as principal vectors, its high hematopathogenicity and its importance in the epizootiology of the disease. In addition, the predominance of *T. congolense* is believed to be due to cattle exposure to *Glossina morsitans* and *Glossina pallipides* which appeared in this area several decades ago and which are efficient in transmission of *T. congolense* and *T. vivax* [19,20]. Furthermore, mix infections (with prevalence and PCV) such as: *T. congolense* + *T. vivax* + *T. brucei* (prevalence=8.78%; PCV=28.77%) and *T. congolense* + *T. vivax* (prevalence=8.11%; PCV=29.08%) were most prevalent and less hematopathologic while *T. congolense + T. brucei* (1.35%; 23%) was less prevalent and most hematopathologic in this area. Hence, diagnosis following such single (*T. congolense*) and mix (*T. congolense + T. brucei*) infections should be given quick veterinary attention.

With regards to the PCV values obtained from this experiment in Alme, an assumption can be made on the infection status of the animals with the various trypanosome species. Anemia, which is best measured by PCV, remains one of the key-indicators of trypanosomiasis in cattle [21]. A high fraction (33.10%) of these infected animals falls within the PCV interval of (28-31%) while the least fraction (3.40%) within 40-43% hence from Figure-2 it shows that highest occurrences of trypanosomes was prominent in animals with PCV ≤31% and lowest as from >31%; which is beyond the normal PCV value as propounded by Kelly, W.R. [22] and slightly greater than that stipulated by Girma *et al*. [23]. This finding can be partly explained by the fact that the microhematocrit buffy coat technique (BCT) of detecting trypanosomes in blood is more sensitive since it detects slight infections than that of direct smear examination [24]. Furthermore, high PCV values in infected cases can also be explained by the abundant pasture land for the available herds at the time of the study.

The microhematocrit BCT is also advantageous in that it indicates the general condition of the animal by PCV determination. However in areas where other anemia-causing factors prevail, PCV alone might not be the sole indicator of choice for detecting trypanosome infections. PCV can also be lowered equally by other anemia causing factors such as tick infestation, helminthosis, hemoparasitosis (other than trypanosomiasis) and nutritional deficiencies in the area [4].

Some breeds of cattle have been shown to have a degree of innate trypanotolerance against species of trypanosomes like the Daoyo/Namchi (*Bos taurus*) [5]. Even though the Brahman/Bokolo breed registered lowest prevalence as compared to others, this finding could be due to small sample size. The present study reveals that the lowest prevalence was from Brahman race as compared to others such as the red Fulani, white Fulani and the Gudali, but this disagrees with the finding of Mbahin *et al*. [25] in the same study area, where the smallest infection rate was rather recorded with the Gudali and did not include the Brahman race in the study.

The effect of trypanosomiasis on the mean PCV with respect to sex of the animals was also checked and it revealed that, mean PCV of male cattle (25.64±5.08 SD) was significantly lower than that of females (30.82±4.94 SD) (p<0.05). This indicates that male animals were more susceptible to infection than females and this agrees with results obtained by Orenge *et al*. [26] in which female N’Dama × Kenya-Boran breed was more trypanotolerant than the male under natural trypanosome challenge. This finding can be explained that, in this area, the breeding system used is a natural mating system with bulls in a competitive chase for the few females all year round, with no supplementary feeding hence stress from such activity exposes the males to infections of this nature than females as reflected in their infection rates. Furthermore, males with a high prevalence recorded low PCV values due to the presence of the pathogen in their systems as compared to females. The trend of infection rate with mean PCV was also judged from a scatter-linear plot, which resulted in decrease PCV, with increase infection rate of trypanosomiasis. This relation is similar to the previous finding of Yehunie *et al*. [27].

## Conclusion

Cattle breeds irrespective of being trypanotolerant or not, which had naturally co-existed with the parasite in single and mix infection states, for several millennia in a vector dense and rich pastural zone such as Alme of the Adamaoua Plateau of Cameroon, do not show a significant difference with parasitism (single or mix infections), resist alterations in PCV, but remains productive despite the relative challenges such as emaciation, loss of body weight and poor body condition. Anemia and other related risk factors such as poor body condition and weight loss will not be reliable signs for trypanosomiasis diagnosis in a zone where other blood dwelling parasites prevail. However, such vital signs should always be followed by buffy coat examination, which is affordable and reliable before any treatment.

## Authors’ Contributions

MA and VKP designed the Study. MA and SLS carried out the study on the field. SLS analyzed the data and prepared the manuscript. MA and VKP reviewed the manuscript. All authors participated in scientific discussion. All authors read and approved the final manuscript.
